# Determination of gastric atrophy with artificial intelligence compared to the assessments of the modified Kyoto and OLGA classifications

**DOI:** 10.1002/jgh3.12810

**Published:** 2022-08-26

**Authors:** Yasuhiro Kodaka, Seiji Futagami, Yoshiyuki Watanabe, Satoki Shichijo, Noriya Uedo, Hiroyuki Aono, Kumiko Kirita, Yusuke Kato, Nobue Ueki, Shuhei Agawa, Hiroshi Yamawaki, Katsuhiko Iwakiri, Tomohiro Tada

**Affiliations:** ^1^ Division of Gastroenterology Nippon Medical School Tokyo Japan; ^2^ Department of Gastrointestinal Oncology Osaka International Cancer Institute Osaka Japan; ^3^ AI Medical Service Inc. Tokyo Japan; ^4^ Tada Tomohiro Institute of Gastroenterology and Proctology Saitama Japan

**Keywords:** artificial intelligence, gastric atrophy, Kyoto classification, Operative Link on Gastritis Assessment classification

## Abstract

**Background and Aim:**

Gastric atrophy is a precancerous lesion. We aimed to clarify whether gastric atrophy determined by artificial intelligence (AI) correlates with the diagnosis made by expert endoscopists using several endoscopic classifications, the Operative Link on Gastritis Assessment (OLGA) classification based on histological findings, and genotypes associated with gastric atrophy and cancer.

**Methods:**

Two hundred seventy *Helicobacter pylori*‐positive outpatients were enrolled. All patients' endoscopy data were retrospectively evaluated based on the Kimura‐Takemoto, modified Kyoto, and OLGA classifications. The AI‐trained neural network generated a continuous number between 0 and 1 for gastric atrophy. Nucleotide variance of some candidate genes was confirmed or selectively assessed for a variety of genotypes, including the *COX‐2*1195, *IL‐1β* 511, and *mPGES‐1* genotypes.

**Results:**

There were significant correlations between determinations of gastric atrophy by AI and by expert endoscopists using not only the Kimura‐Takemoto classification (*P* < 0.001), but also the modified Kyoto classification (*P* = 0.046 and *P* < 0.001 for the two criteria). Moreover, there was a significant correlation with the OLGA classification (*P* = 0.009). Nucleotide variance of the *COX‐2*, *IL‐1β*, and *mPGES‐1*genes was not significantly associated with gastric atrophy determined by AI. The area under the curve values of the combinations of AI and the modified Kyoto classification (0.746) and AI and the OLGA classification (0.675) were higher than in AI alone (0.665).

**Conclusion:**

Combinations of AI and the modified Kyoto classification or of AI and the OLGA classification could be useful tools for evaluating gastric atrophy in patients with *H*. *pylori* infection as the risk of gastric cancer.

## Graphical abstract

Gastric atrophy determined by artificial intelligence (AI) correlates with the diagnosis using the Kimura‐Takemoto and modified Kyoto classifications by expert endoscopists, and the Operative Link on Gastritis Assessment (OLGA) classification based on histological findings. Combinations of AI and the modified Kyoto classification or of AI and the OLGA classification could be useful tools for evaluating gastric atrophy in patients with H. pylori infection as the risk of gastric cancer.

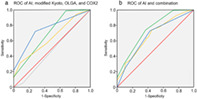

## Introduction

In recent years, artificial intelligence (AI) with deep learning has made remarkable progress in various medical fields, especially as a system to screen medical images. There are reportedly many roles for AI with deep learning in medical practice, including radiation oncology,^1^ skin cancer classification,^2^ diabetic retinopathy,^3^ and digestive tract endoscopy, especially colonoscopy.^4^, ^5^, ^6^ For gastrointestinal diagnosis, in particular, AI with deep learning has been proposed for use in gastric cancer,^7^ esophageal cancer,^8^ small‐bowel angioectasia,^9^ detections of erosions and ulcerations in wireless capsule endoscopy images,^10^ and *Helicobacter pylori* infection status.^11^

Endoscopists combine their knowledge of the spectrum of endoscopic appearances such as the precancerous lesions of intestinal metaplasia and gastric atrophy. Unfortunately, during gastroduodenal endoscopy, both lesion detection and image assessment of detected lesions to predict histology are subject to substantial operator dependence. However, there are no data currently available about the usefulness of AI in recognizing gastric atrophy as a precancerous lesion. Gastric atrophy, intestinal metaplasia, enlarged‐fold appearance, and inflammation within the gastric body have been reported as risk factors for the development of gastric cancer.^12^, ^13^, ^14^ In Japan, the new Kyoto Global Consensus Meeting on *H*. *pylori* gastritis proposed that the risk of *H*. *pylori*‐infected gastritis should be determined based on the extent of gastric atrophy.^15^ Also, the Operative Link on Gastritis Assessment (OLGA) system incorporated the semiquantitative scoring established by the Sydney System and the subsequent Atrophy International Club Guidelines and expresses the extent/location of atrophic/metaplastic lesions in terms of gastritis staging.^16^ In addition, some studies have reported that *COX‐2* and *IL‐1 β* genotypes were significantly associated with gastric atrophy. Therefore, we tried to clarify whether *COX‐2*1195, *IL‐1β* 511, and *mPGES‐1* genotypes, which have been reported to be associated with gastric cancer, were also associated with gastric atrophy determined by AI.

In this study, we compared the determination of gastric atrophy by AI with atrophy diagnosed by expert endoscopists using the modified Kyoto classification, the histological OLGA classification, and the *COX‐2*1195, *IL‐1β* 511, and *mPGES‐1* genotypes.

## Materials and methods

### Patients

This study enrolled 270 *H*. *pylori*‐positive outpatients, who either presented with upper gastrointestinal symptoms or for a periodical check‐up between March 2010 and May 2015. The gastroenterologists performing the esophagogastroduodenoscopy (EGD) for this study had at least 10 years of experience with the procedure. Exclusion criteria included renal failure, liver cirrhosis, and a history of malignant disease. Patients with previous gastroduodenal surgery, diabetes mellitus, and recent use of non‐steroidal anti‐inflammatory drugs (NSAIDs) at the time of endoscopy were also excluded. *H*. *pylori* infection was determined by the ^13^C‐urea breath test or histological identification. Endoscopic assessments were performed on all enrolled patients before eradication therapy. Written informed consent was obtained from all subjects prior to undergoing upper gastrointestinal endoscopy. The study protocol was approved by the Ethics Review Committee of Nippon Medical School Hospital (490‐31‐19). The study was conducted ethically in accordance with the World Medical Association Declaration of Helsinki.

### The Kimura‐Takemoto classification as a reference standard

We used two grades of gastric atrophy diagnosed by expert endoscopists using the Kimura‐Takemoto classification (C‐I and C‐II *vs* C‐III, O‐I, O‐II, and O‐III) as the reference standard (Table 1).^11^

**Table 1:** Characteristics of patients as the reference standard

	Total	Male	Female	*P* value
Numbers of the patients	270	132	138	
Age (mean ± SD, years)	59.5 ± 13.9	59.5 ± 13.5	59.6 ± 14.4	0.97
*H*. *pylori* positivity (%)	100	100	100	1
Kimura‐Takemoto classification				
C‐I, C‐II (*n*)	54	23	31	0.30
C‐III, O‐I, O‐II, O‐III (*n*)	216	109	107	

### Assessment of endoscopic appearance based on the modified Kyoto classification

Retrospective analysis was carried out on data from prior endoscopy. All enrolled patients had previously undergone endoscopy. Atrophic fold and regular arrangement of collecting venules (RAC) at the gastric angle were scored retrospectively for each patient based on the modified Kyoto classification as either 0: absent, or 1: present.^17^ Gastric atrophy was scored as either 0: the atrophy distribution was localized to the gastric antrum and was not observed endoscopically, or 1: the atrophy distribution was present in the gastric antrum and body.

### Evaluating the extent of gastric atrophy using the OLGA classification

We also retrospectively assessed the extent of gastric atrophy based on the OLGA staging system, using biopsy specimens from the lesser curvature of the antrum and the greater and lesser curvatures of the gastric body. The OLGA system incorporates the semiquantitative scoring systems established by the Sydney System and the subsequent Atrophy International Club Guidelines and expresses the extent/location of atrophic/metaplastic lesions in terms of gastritis staging.^16^ The OLGA stage results from a combination of the extent of atrophy scored histologically and the topography of atrophy identified through biopsy mapping.

### Training algorithm

To construct an AI‐based diagnostic system, we used a state‐of‐the‐art deep neural architecture, Res Net (https://www.cv‐foundation.org/openaccess/content_cvpr_2016/papers/He_Deep_Residual_Learning_2016_paper.pdf), which was developed by Szegedy *et al*. Res Net34 is a convolutional neural network (CNN) consisting of 34 layers.

### Preparation of training

The inclusion criteria were images with standard white light. The exclusion criteria were any images that were magnified, as well as poor quality images resulting from insufficient air insufflation, post‐biopsy bleeding, halation, blurring, defocus, or mucus.

### Training image sets

After selection, 11 497 images were collected for 4373 with gastric atrophy and 7124 without as a training image data set. This set was determined by expert endoscopists at the Osaka International Cancer Institute and considered as the gold standard for AI diagnosis. All EGD procedures used a standard endoscope (GIF‐H290Z) and a standard endoscopic video system. 4294 images (downward view), 4003 images (retroflex view), and 3200 images (antrum) were evaluated. During the procedure, the entire stomach was observed, and images of all parts were captured with white light images. The chromoendoscopy, narrow band imaging, and poor‐quality images were excluded. Briefly, endoscopic images were classified into three categories according to the gastric location: “antrum,” “gastric body (antegrade view),” and “gastric body (retroflex view).” The AI system distinguished six sorting classes from the images. These six classes are based on the presence or absence of atrophy for each region or view (antrum, body antegrade view, and body retroflex view) and cited from a previous study.^18^

### Test image sets and evaluation algorithm

From 270 *H*. *pylori*‐positive patients, 7724 images were evaluated by the CNN from Nippon Medical School Hospital. The images were taken with various endoscopes (GIF‐H290Z (51/270), GIF‐H260Z (87/270), GIF‐H260 (96/270), GIF‐XP260N (36/270)). All of the test images were captured with white light images. The definition of gastric atrophy in the test image sets was determined by AI, based on the training image sets.

The case was classified as “severe atrophy” when both the body (antegrade view) and the body (retroflex view) were judged as atrophic. “Moderate atrophy” was assigned when the body (antegrade view) was non‐atrophic and the body (retroflex view) was atrophic. “Mild atrophy” was assessed for cases when both the body (antegrade view) and the body (retroflex view) were judged as non‐atrophic. Besides, we divided into two grades “none to mild” atrophy and “moderate to severe” atrophy to compare with another assessment.

### Genotyping

DNA was extracted from peripheral blood leukocytes or gastric biopsy specimens using a commercially available kit (Ambion, Austin, TX, USA). We have developed or optimized the following assays for genetic variation. Genotypes were confirmed or selectively assessed for a variety of genotypes, including (*COX‐2*1195 A‐>G; rs: 689466), (*IL‐1β* 511T‐>C; rs:16944), and (*mPGES‐1* C‐>A; rs: 2302821) genotypes, using an ABI 7500 Fast. Gene polymorphisms were determined according to methods used in other studies as follows. Real‐time polymerase chain reaction using Taq Man chemistries (Applied Biosystems, Foster City, CA, USA) was used to determine alleles present in each sample. The real‐time polymerase chain reactions were performed in an Applied Biosystems 7500 Fast machine (Applied Biosystems). The Taq Man primer probe assays for *COX‐2* SNPs 1195 A‐>G (rs: 689466; C‐2517145‐20), *IL‐1β* SNPs 511T‐>C (rs:16944; C‐1839943‐10), and *mPGES‐1* SNPs C‐>A (rs: 2302821; C‐15758147‐10) were purchased from Applied Biosystems. Briefly, each 10 μL of reaction volume consisted of 5 μL TaqMan Genotyping Master Mix (Applied Biosystems), 0.25 μL of a 40× primer‐probe assay mix (Applied Biosystems), 3.75 μL H_2_O and 1 μL (10 ng) genomic DNA. The amplification conditions were; 95°C, 10 min; 90 cycles of 95°C, 15 s; 58°C, the 30s; followed by 60°C, 1 min for *COX‐2* and 95°C, 10 min; 40 cycles 95°C, 15 s; 60°C, 60s; followed by 60°C, 1 min for *IL‐1β* and *mPGES‐1*. The data were analyzed using automated software (SDS 2.1, Applied Biosystems) to determine the genotype of each sample.

### Statistical analysis

The Chi‐squared test was used to compare the Kimura‐Takemoto and OLGA classifications diagnosed by expert endoscopists with the degree of gastric atrophy determined by the AI. The Chi‐squared test was also used to compare genotypes with the degree of gastric atrophy determined by the AI. In addition, Fisher's exact test was used to compare the modified Kyoto classification diagnosed by expert endoscopists with the degree of gastric atrophy determined by the AI. Receiver operating characteristic (ROC) curves were created by plotting sensitivity, as a proportion, *versus* (1‐specificity), as a proportion. A larger area under the ROC curve indicated better diagnostic performance. We plotted ROC curves of the severity of gastric atrophy determined by AI alone, *COX‐2* genotype alone, and one each for the modified Kyoto and OLGA classifications determined by expert endoscopists. The ROC curve for the modified Kyoto classification was plotted using the *Z*‐Score, the calculated sum of the RAC and atrophic fold. The combination ROC curves, of both the AI and modified Kyoto classification and the AI and OLGA classification, were plotted by adding each *Z*‐Score. Statistical analyses were carried out with SPSS v26 (IBM Corp, Arkmont, NY, USA).

## Results

### Characteristics of the reference standard

In total, 270 *H*. *pylori*‐positive patients were enrolled (mean age: 59.5 ± 13.9 years; sex: 132 male/138 female; Table 1). We divided enrolled patients (*n* = 270) into two grades of gastric atrophy assessed by expert endoscopists using the Kimura‐Takemoto classification ([C‐I, C‐II: *n* = 54] and [C‐III, O‐I, O‐II, O‐III: *n* = 216]) as the reference standard (Table 1) whereas, AI diagnosed gastric atrophy in two grades, “none to mild” (*n* = 96) and “moderate to severe” (*n* = 174; Table 2).

**Table 2:** Comparison of the degree of gastric atrophy: artificial intelligence (AI) *versus* expert endoscopists using the Kimura‐Takemoto classification

	The degree of gastric atrophy determined by AI		
	None to mild	Moderate to severe	Total
Diagnosis by expert endoscopists using the Kimura‐Takemoto classification			
C‐I, C‐II	31	23	54
C‐III, O‐I O‐II, O‐III	65	151	216
Total	96	174	270

There was a significant correlation (*P* < 0.001, Chi‐squared test) between the degree of gastric atrophy determined by AI and the experts using the Kimura‐Takemoto classification.

### *Comparison of the degree of gastric atrophy: AI* versus *expert endoscopists using the Kimura‐Takemoto classification*

We compared the AI's diagnostic performance with the grade of gastric atrophy assessed by expert endoscopists using the Kimura‐Takemoto classification. 31 cases of (C‐I, C‐II: *n* = 54) were assessed with “none to mild” gastric atrophy by AI, and 151 cases of (C‐III, O‐I, O‐II, O‐III: *n* = 216) were diagnosed with “moderate to severe” gastric atrophy by AI. There was a significant correlation (*P* < 0.001) between the two assessments (Table 2). The accuracy of the AI's diagnosis compared to the Kimura‐Takemoto classification was 67.4% (182/270).

### *Comparison of the degree of gastric atrophy: AI* versus *expert endoscopists using the modified Kyoto classification*

On the modified Kyoto classification, 24 cases showed an atrophic fold in the greater curvature of the gastric body. 20 of these cases were diagnosed as “moderate to severe” gastric atrophy by AI (specificity: 20/24; 83.3%). In contrast, 246 cases were considered atrophic fold‐negative in the greater curvature of the gastric body on the modified Kyoto classification. Of these, 92 cases were assessed with “none to mild” gastric atrophy by AI (sensitivity: 92/246; 37.4%) (Table 3). There was a significant correlation (*P* = 0.046) between the two assessments (Table 3).

**Table 3:** Comparison of the degree of gastric atrophy: artificial intelligence (AI) *versus* expert endoscopists using the modified Kyoto classification

	The degree of gastric atrophy determined by AI		
	None to mild	Moderate to severe	Total
Diagnosis by expert endoscopists using the modified Kyoto classification			
Atrophic fold (−)^†^	92	154	246
Atrophic fold (+)^†^	4	20	24
Total	96	174	270

	The degree of gastric atrophy determined by AI		
	None to mild	Moderate to severe	Total
Diagnosis by expert endoscopists using the modified Kyoto classification			
RAC (+)^‡^	15	5	20
RAC (−)^‡^	81	169	250
Total	96	174	270

† There was a significant correlation (*P* = 0.046, Fisher's exact test) between an assessment of atrophic fold using the modified Kyoto classification and the degree of gastric atrophy determined by AI.

‡ There was a significant correlation (*P* < 0.001, Fisher's exact test) between an assessment of RAC using the modified Kyoto classification and the degree of gastric atrophy determined by AI.

We also compared the grade of gastric atrophy by AI to assessments of RAC at the gastric angle on the modified Kyoto classification. The expert endoscopists identified 20 RAC‐positive patients, and 15 of these were assessed by AI as “none to mild” gastric atrophy (sensitivity: 15/20; 75.0%). In contrast, the experts assessed 250 RAC‐negative cases, and 169 of these were determined as “moderate to severe” gastric atrophy by AI (specificity: 169/250; 67.6%). There was a significant correlation (*P* < 0.001) between the degree of gastric atrophy determined by AI and the grade of gastric atrophy according to the existence of RAC using the modified Kyoto classification (Table 3). The accuracy of gastric atrophy determined by AI compared to the RAC using the modified Kyoto classification was 68.1% (184/270).

### *Comparison of the degree of gastric atrophy: AI* versus *histological assessment using the OLGA classification*

Based on the OLGA classification as histological assessment, there were 12 stages 0, 52 stage I, 37 stages II, 23 stage III, and 11 stage IV cases of gastric atrophy in this study. 135 cases could not be histologically diagnosed. Stage 0 and I of the OLGA classification were a total of 64 cases, and 34 of these would correspond to the “none to mild” gastric atrophy by AI. Stage II, III, and IV of the OLGA classification were a total of 71 cases, and 50 of these would correspond to the **“**moderate to severe” gastric atrophy by AI (Table 4). There was a significant correlation (*P* = 0.009) between the stage of gastric atrophy diagnosed using the OLGA classification and the degree of gastric atrophy determined by AI (Table 4). The accuracy of gastric atrophy determined by AI and compared to the OLGA classification was 62.2% (84/135).

**Table 4:** Comparison of the degree of gastric atrophy: AI *versus* histological assessment using the OLGA classification

	The degree of gastric atrophy determined by AI		
	None to mild	Moderate to severe	Total
Diagnosis by histological assessment using the OLGA classification			
Stage 0, I	34	30	64
Stage II, III, IV	21	50	71
Total	55	80	135

There was a significant correlation (*P* = 0.009, Chi‐squared test) between the stage of gastric atrophy diagnosed using the OLGA classification and the degree of gastric atrophy determined by AI.

AI, artificial intelligence; OLGA, Operative Link on Gastritis Assessment.

### Comparison of COX‐21195, IL‐1β 511, and mPEGS‐1 genotypes with the degree of gastric atrophy determined by AI

We investigated whether the degree of gastric atrophy determined by AI was significantly associated with three genotypes correlated with gastric atrophy and cancer. The *COX‐2*1195G‐carrier genotype was not significantly (*P* = 0.796) associated with the degree of gastric atrophy determined by AI (Table 5). The *IL‐1β* 511 TT genotype was also not significantly (*P* = 0.850) associated with the degree of gastric atrophy determined by AI (Table 5). Finally, the *mPGES‐1* genotype was also not significantly (*P* = 0.759) associated with the degree of gastric atrophy determined by AI (Table 5).

**Table 5:** Comparison of *COX‐2*1195, *mPGES‐1*, and *IL‐1β* 511 genotypes with the degree of gastric atrophy determined by artificial intelligence (AI)

	The degree of gastric atrophy determined by AI		
	None to mild	Moderate to severe	Total
*COX‐2*1195			
AG, GG	56	106	162
AA	34	60	94
Total	90	166	256
*IL‐1β* 511			
TT	18	35	53
CT, CC	76	139	215
Total	94	174	268
*mPGES‐1*			
AA	18	30	48
AG, GG	77	142	219
Total	95	172	267

The degree of gastric atrophy determined by AI was not significant (*P* = 0.796, *P* = 0.759, and *P* = 0.850; Chi‐squared test) associated with *COX‐2*1195, *IL‐1β* 511, and *mPGES‐1* genotypes.

### Comparison of area under the curve values

**Figure 1:**
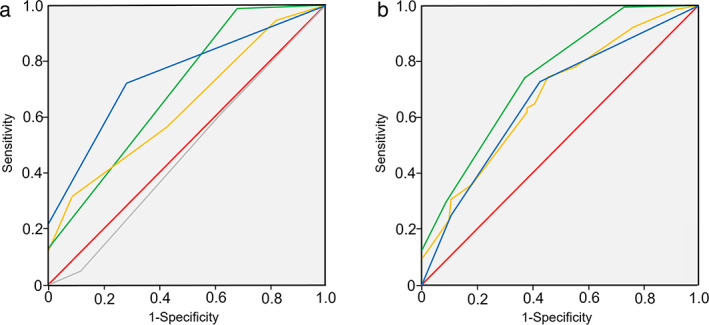
Comparison of area under the curve (AUC) values. (a) Receiver operating characteristic (ROC) curve among four groups, plotting the degree of gastric atrophy determined by artificial intelligence (AI), the Operative Link on Gastritis Assessment (OLGA) classification, the modified Kyoto classification, and the *COX2* 1195 genotype. The AUC values were 0.748 for AI, 0.636 for the OLGA classification, 0.696 for the modified Kyoto classification, and 0.471 for the *COX2* 1195 genotype. 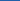, AI; 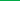, modified Kyoto; 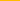, OLGA; 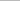, COX2; 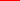, reference line. (b) ROC curve among three groups, plotting the degree of gastric atrophy determined by AI, a combination of AI and the OLGA classification, and a combination of AI and the modified Kyoto classification. 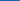, AI; 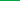, combination of AI and modified Kyoto; 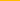, combination of AI and OLGA; 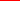, reference line. The AUC values were 0.665 for AI, 0.675 for the combination of AI and the OLGA classification, and 0.746 for the combination of AI and the modified Kyoto classification.

To compare the differences between determinations of gastric atrophy by AI, the modified Kyoto classification, the OLGA classification, and the *COX2* 1195 genotype, we plotted ROC curves for the four groups (Fig. 1a). The area under the curve (AUC) values were 0.748 for AI, 0.696 for the modified Kyoto classification, 0.636 for the OLGA classification, and 0.471 for the *COX2* 1195 genotype.

In addition, to compare the differences between determinations of gastric atrophy by AI alone, by AI and the modified Kyoto classification together, and by AI and the OLGA classification together, we plotted ROC curves for the three groups (Fig. 1b). The AUC values were 0.665 for AI alone, 0.746 for the combination of AI and the modified Kyoto classification, and 0.675 for the combination of AI and the OLGA classification.

## Discussion

The major findings of this study are (i) There was a significant correlation between the degree of gastric atrophy determined by AI and that diagnosed by expert endoscopists using the Kimura‐Takemoto classification (*P* ≤ 0.001). (ii) There were significant correlations between the degree of gastric atrophy determined by AI and that diagnosed by expert endoscopists using the RAC (*P* ≤ 0.001) and atrophic fold (*P* = 0.046) criteria of the modified Kyoto classification. (iii) There was a significant correlation between the degree of gastric atrophy determined by AI and that diagnosed using the histological OLGA classification (*P* = 0.009). (iv) The AUC values of the combination of AI and the modified Kyoto classification (AUC: 0.746) and of AI and OLGA classification (AUC: 0.675) were higher than that in AI alone (AUC: 0.665).

We constructed an AI‐based diagnostic system to detect gastric atrophy as a risk factor for gastric cancer using many endoscopic images. To the best of our knowledge, this is the first report estimating the ability of a CNN to detect gastric atrophy from endoscopic images. Since the severity and extent of atrophic gastritis are well established as indicators of increased risk for developing gastric cancer,^19^, ^20^, ^21^ evaluation of gastric atrophy with AI could play an important role in gastric cancer screening. In this study, we focused on the extent of gastric atrophy as the risk for the advance of gastric cancer and compared determinations by AI with those made by expert endoscopists. The assessments of gastric atrophy by AI correlated significantly with those of the experts using the Kimura‐Takemoto (*P* < 0.001) and modified Kyoto (*P* < 0.001) classifications. Looking more closely, the sensitivity of AI diagnosis using the RAC criteria of the modified Kyoto classification (15/20: 75.0%) was higher than that using atrophic fold criteria (92/246: 37.4%). There were no data to explain this difference. In addition, AI diagnosis of patients with mild gastric atrophy did not correspond to the endoscopic classification of *H*. *pylori*‐positive gastritis. However, AI diagnosis did correspond to RAC positivity in patients with moderate and severe gastric atrophy according to the negative predictive value (169/174: 97.1%). The presence of gastric mucous, halation, and shadow on test images could have influenced the discrepancy between AI diagnosis and endoscopic classification with the Kimura‐Takemoto and modified Kyoto classifications (Figure S1, Supporting information). Better endoscopic pictures using basic techniques are needed to avoid these problems. Precise AI diagnosis may require improvements in these techniques.

In this study, gastric atrophy determined by AI was significantly correlated with the extent of gastric atrophy based on the OLGA classification, as shown in Table 4. Comparison of the AUC values of determinations by AI *versus* the OLGA classification was shown in Figure 1a. The degree of gastric atrophy determined by AI had a significantly higher AUC value than that by the OLGA classification. Similarly, gastric atrophy determined by AI was significantly correlated with the assessment using the modified Kyoto classification, as shown in Table 3. We compared the AUC value for the AI determination with that for the modified Kyoto classification, as shown in Figure 1a. Thus, the AI determination showed a significantly higher AUC value compared to that for the modified Kyoto classification, representing the endoscopic evaluation. Moreover, the combination of AI and the modified Kyoto classification exhibited a higher AUC value for the determination of gastric atrophy than that of AI alone, suggesting that the AI system can serve to support the endoscopic diagnosis of gastric atrophy.

Furuta *et al*. have reported that in *H*. *pylori*‐infected gastritis, the *IL‐1β* 511T/T genotype was associated with the gastric atrophy score.^22^ However, we did not find a significant relationship between the *IL‐1β* genotype and the degree of gastric atrophy determined by AI. In addition, although Achyut *et al*. have reported that *COX‐2*765 C allele carriers had a low risk for gastric atrophy,^23^ we did not observe a significant relationship between the *COX‐2* genotype and gastric atrophy determined by AI. Single nucleotide polymorphism of *COX‐2*1195 is one of the factors related to gastric atrophy. However, the AUC of *COX‐2*1195 (0.471) did not show enough detection power.

This study has several limitations. First, it was a single‐center retrospective study. We think that the outcomes of this study are reliable because verification was performed prospectively. Second, we used only high‐quality endoscopic images for the training of the AI, but the test images were taken by various endoscopes, including low‐quality ones. Thus, we are not sure whether CNN can diagnose gastric atrophy using low‐quality images, such as those that show halation, mucus, or those that are out of focus. Third, this study enrolled only patients who were positive for *H*. *pylori*. The number of cases presenting after *H*. *pylori* eradication is currently increasing in Japan.

Further studies will be needed to investigate whether the evaluation of gastric atrophy using the combination of AI and endoscopic classifications such as the modified Kyoto classification will be superior to either AI or endoscopic determinations alone in *H*. *pylori*‐negative and *H*. *pylori*‐eradicated patients because most people are now free from *H*. *pylori* infection in Japan. Furthermore, our findings from this retrospective study will require validation in prospective studies.

Taken together, the degree of gastric atrophy determined by AI correlated significantly with the degree of atrophy diagnosed by expert endoscopists using the Kimura‐Takemoto and modified Kyoto classifications, as well as the OLGA classification based on histological findings. Combinations of AI and the modified Kyoto classification or of AI and the OLGA classification may be useful tools to predict the development of gastric cancer through evaluations of gastric atrophy.

## Supporting information


**Figure S1**. (A) Gastric antrum. The diagnosis by expert endoscopists was the presence of gastric atrophy, and the determination by AI was also the presence of gastric atrophy. Correct case. (B) Gastric antrum. Although the diagnosis by expert endoscopists was the presence of gastric atrophy; the determination by AI was none. Incorrect case. The cause for the false‐negative could be the halation and shadow in the photo. (C). Gastric body (retroflex view). The diagnosis by expert endoscopists was the presence of gastric atrophy and determination by AI was also the presence of gastric atrophy. Correct case. (D) Gastric body (retroflex view). Although the diagnosis by expert endoscopists was the presence of gastric atrophy; the determination by AI was none.
Incorrect case. The cause for the false‐negative could be the halation and shadow in the photo. (E) Gastric body (antegrade view). The diagnosis by expert endoscopists was the presence of gastric atrophy and the determination by AI was also the presence of gastric atrophy. Correct case. (F). Gastric body (antegrade view). Although the diagnosis by expert endoscopists was the presence of gastric atrophy; the determination by AI was none. Incorrect case. The cause for the false negative could be the shadow in the photo.

## Data Availability

The data used to support the findings of this study are included in the article.
