# Supplementary figures and images for: Determination of gastric atrophy with artificial intelligence compared to the assessments of the modified Kyoto and OLGA classifications
Source: JGH Open. 2022 Aug 26;6(10):704–10. doi: 10.1002/jgh3.12810 (PMC9575326; doi:10.1002/jgh3.12810)

**Supplementary Figure**


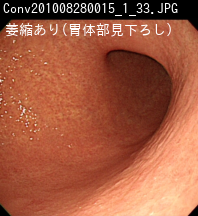


E


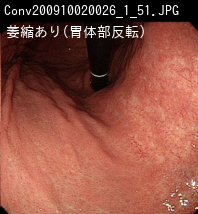


C


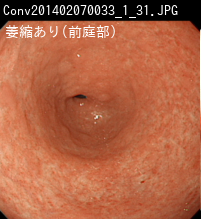


A


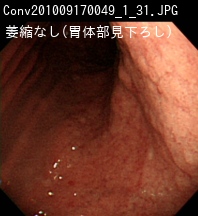


F


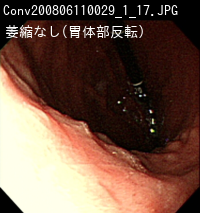


D


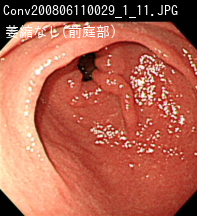


B

Supplement: Supplementary file 1 — Figure S1. (A) Gastric antrum. The diagnosis by expert endoscopists was the presence of gastric atrophy, and the determination by AI was also the presence of gastric atrophy. Correct case. (B) Gastric antrum. Although the diagnosis by expert endoscopists was the presence of gastric atrophy; the determination by AI was none. Incorrect case. The cause for the false‐negative could be the halation and shadow in the photo. (C). Gastric body (retroflex view). The diagnosis by expert endoscopists was the presence of gastric atrophy and determination by AI was also the presence of gastric atrophy. Correct case. (D) Gastric body (retroflex view). Although the diagnosis by expert endoscopists was the presence of gastric atrophy; the determination by AI was none. Incorrect case. The cause for the false‐negative could be the halation and shadow in the photo. (E) Gastric body (antegrade view). The diagnosis by expert endoscopists was the presence of gastric atrophy and the determination by AI was also the presence of gastric atrophy. Correct case. (F). Gastric body (antegrade view). Although the diagnosis by expert endoscopists was the presence of gastric atrophy; the determination by AI was none. Incorrect case. The cause for the false negative could be the shadow in the photo. [file JGH3-6-704-s001.docx]
